# Differences in Attitudes Toward Reading: A Survey of Pupils in Grades 5 to 8

**DOI:** 10.3389/fpsyg.2018.02773

**Published:** 2019-01-11

**Authors:** Pascale Nootens, Marie-France Morin, Denis Alamargot, Carolina Gonçalves, Michèle Venet, Anne-Marie Labrecque

**Affiliations:** ^1^Research Chair in Reading and Writing Learning in Young Children, Faculty of Education, Sherbrooke University, Sherbrooke, QC, Canada; ^2^Human and Artificial Cognitions (CHArt) Laboratory, University of East Paris Créteil, Paris, France; ^3^Instituto Politécnico de Lisboa, Lisbon, Portugal; ^4^Centro Interdisciplinar de Ciências Sociais (CICS), Faculdade de Ciências Sociais e Humanas (FCSH), Universidade NOVA de Lisboa, Lisbon, Portugal

**Keywords:** leisure reading, academic reading, reading attitude, pupils, students, elementary school, secondary school, Quebec (Canada)

## Abstract

Recent research on literacy has highlighted the impact of affective factors on learning to read. Among these factors, attitudes toward reading have been clearly shown to influence the development of reading skills and academic success. Nevertheless, differences in children’s attitudes across schooling have yet to be properly documented, especially for the French language and the transition between elementary and secondary education. In this cross-sectional study, our goal was to gauge the attitudes of French-speaking pupils across this transitional period. We therefore administered a computer-based questionnaire to 469 pupils in Grades 5 to 8 in Quebec (Canada), to gather their views about leisure reading and academic reading. Results showed that their stated attitudes toward reading remained stable across the final 2 years of elementary school, as well as across the first 2 years of middle school, but differences were observed for the transition from one education level to the next, with stated attitudes toward reading being less positive in the latter. This effect, which was observed for both leisure and academic reading, concerned girls and boys alike. We discuss possible explanations for these differences in reading attitudes at this juncture in children’s schooling.

## Introduction

In this cross-sectional study, our goal was to gauge the attitudes of French-speaking pupils toward reading across the transition between elementary and secondary education. For several decades, researchers have sought to gain a better understanding of how children learn to read, the difficulties they may encounter along the way, and the most effective means of avoiding or overcoming these difficulties. Despite the implementation, mainly in North America (National Center for Education Statistics, 2009), of several large-scale intervention programs based on sound data, a substantial proportion of students still fail to attain minimum thresholds for reading literacy (Organisation for Economic Co-operation and Development [OECD], 2015). This stark fact has prompted researchers to examine how learning to read is impacted by affective factors such as literacy interest (Hume et al., 2015), engagement (De Naeghel et al., 2012), motivation (Gambrell et al., 1996; Baker and Wigfield, 1999; Guthrie et al., 1999; Anmarkrud and Braten, 2009; Becker et al., 2010; Marinak and Gambrell, 2010), self-concept (Katzir et al., 2009) and attitude toward reading (McKenna et al., 1995, 2012; Conlon et al., 2006; Martinez et al., 2008; Clark and De Zoysa, 2011; Clark, 2014). The links between reading attitude and achievement are now well documented (Petscher, 2010), especially in the United States, as are changes in attitudes toward reading, at least among elementary school pupils. However, research on attitudes toward reading beyond elementary grades is still scarce (Alexander and Fox, 2011; McKenna et al., 2012), even though the failure of schools in many locations to attend and respond to adolescents’ reading-related motivations makes research on affective factors in reading particularly relevant at the period of transition from childhood to adolescence. Researchers therefore need to examine how reading attitudes change as pupils move from primary to intermediate grades and beyond (Graham et al., 2012). In addition, relevant past research on this topic in certain locations and contexts has still to be uncovered (McKenna et al., 2012). We cannot assume that research findings on this subject (including the affective factors linked to reading) can be directly transferred from one linguistic context to another, and to the best of our knowledge, there have been no recent studies of attitudes to reading during the elementary–secondary transition in French-speaking countries.

In order, therefore, to improve current understanding of attitudes toward reading during the elementary–secondary transition in a French-speaking context, we conducted the present study among pupils in Quebec (Canada). The transition from one level to another takes place between Grades 6 and 7 (i.e., age 12 years) in this province, thus coinciding with the onset of adolescence. *Attitude* has been defined by Fishbein and Ajzen (1975) as a “learned predisposition to respond in a consistently favorable or unfavorable manner with respect to a given object” (p. 6), the term *object* referring to either entities (people, groups) or behaviors (e.g., reading) (Ajzen and Fishbein, 2005).

According to Fishbein and Ajzen (1975)’s conception, attitude is the result of multiple episodes involving the object, each episode shaping the individual’s beliefs about this object (e.g., fun or rather uninteresting). Although relationships between pupils’ attitudes toward reading and the development of reading skills have already been explored (Martinez et al., 2008; Petscher, 2010), changes in these attitudes in the course of their schooling have yet to be properly addressed, especially during the transition from elementary to middle or secondary school. So far, researchers have chosen to focus on a single level of education–either elementary school (McKenna et al., 1995; Hogsten and Peregoy, 1999; Lazarus and Callahan, 2000) or middle school (Ley et al., 1994; McKenna et al., 2012; Kolicì-Vehovec et al., 2014). Although readers aged 9–12 years (i.e., pupils in the upper years of elementary school) are portrayed as avid *consumers* of books, especially for leisure purposes, consumption declines beyond the age of 12 years (Johnsson-Smaragdi and Jönsson, 2006). This age coincides with the entry into secondary education, and also marks the start of adolescence, a period of physical, psychological, environmental and social transition (Lipps, 2005; Benner, 2011; Serbin et al., 2013; Chouinard et al., 2015). Given the acknowledged link between reading habits and attitudes (Ley et al., 1994; Keskin and Bastug, 2014), the elementary–middle/secondary transition should clearly be included in any study of changes in reading attitudes, in order to obtain the most accurate picture possible.

The present study therefore described differences in the attitudes of young readers in Quebec between Grades 5 and 8.

## Previous Research on Reading Attitudes

A number of studies of reading attitudes in elementary or middle school have distinguished between reading goals (academic vs. leisure reading) and looked for sex-related differences. Several studies in this field have also distinguished between digital and print reading, as some authors argue that young people’s literacy experiences, especially from adolescence onward, are not restricted to traditional print settings. Nevertheless, the results of these studies suggest that, as far as academic reading is concerned, reading attitudes remain the same, whatever the medium (McKenna et al., 2012). As far as leisure reading in digital settings is concerned, we reject the very broad conception of digital literacy adopted in some previous studies, such as being on social websites during free time and reading e-mails from friends (see McKenna et al., 2012). Like others (Martin, 2008), we do not consider these particular activities to come under the heading of digital literacy. We therefore chose not to distinguish between digital and print reading when addressing the question of attitudes. Rather, we emulated previous studies on reading attitudes in elementary or middle school that distinguished between reading goals (academic vs. leisure reading) and looked for sex-related differences.

### Reading Attitudes by Grade

Some authors claim that reading attitudes depend on the *nature* or *goal* of the activity (McKenna et al., 1995, 2012; Lazarus and Callahan, 2000; Ivey and Broaddus, 2001), making a distinction between *leisure* (or recreational) reading, when it is the reader who decides when and what to read, and *academic* reading which, for young people, often means reading imposed by a teacher in a school setting (McKenna et al., 2012; Conradi et al., 2013). Most studies in this area, especially cross-sectional ones, have reported a gradual increase in negative attitudes toward reading across elementary school. In a major study conducted in the United States, McKenna et al. (1995) administered the Elementary Reading Attitude Survey (ERAS; McKenna and Kear, 1990) to a stratified sample of 18,185 first to sixth graders drawn from 229 schools in 38 states. This cross-sectional study revealed an overall negative trend in attitudes toward reading across these grades. Whereas pupils starting school had generally positive attitudes, by the end of elementary school, these had been replaced by indifference and even negativity, especially with regard to academic reading. The survey undertaken by Hogsten and Peregoy (1999) among second and sixth graders, this time using the Estes Attitude Scale for Reading, mainly corroborated McKenna et al. (1995)’s results, insofar as attitudes toward reading of whatever kind were not particularly positive at Grade 2, but were clearly negative in Grade 6. When Lazarus and Callahan (2000) replicated McKenna et al. (1995)’s survey, this time among pupils with learning disabilities in special education resource rooms, they found that attitudes toward reading showed a comparable trend between Grades 1 and 5.

Surveys conducted among older children, such as the survey undertaken by McKenna et al. (2012), have reported similar differences in students’ positive attitudes toward reading across middle school. In this cross-sectional study, McKenna et al. (2012) asked 4,491 American middle-school pupils in Grades 6 to 8 to complete an adapted version of the ERAS (McKenna and Kear, 1990)^1^. The authors observed increasingly negative attitudes toward reading, with attitudes toward academic reading appearing to be more stable across grades–but more negative–than those toward leisure reading. In their longitudinal study, Ley et al. (1994) had earlier explored the nature of attitude change among American middle-school pupils, specifically exploring the values placed upon *voluntary* reading for three distinct purposes, namely individual development, utilitarian ends, or enjoyment. They administered the Teale–Lewis Reading Attitude Scales to 164 pupils on three occasions (Grades 6, 7, and 8), in order to disentangle the cognitive, affective and conative aspects of their reading attitudes. Although the authors observed a gradual deterioration in attitudes from Grades 6 to 8, they found that the relative values attributed to reading goals remained stable across this period, with students reading mainly to do well at school, rather than for individual development or enjoyment. Their results therefore foreshadowed McKenna et al. (2012)’s main finding that middle-school readers’ attitudes toward academic reading are generally negative, but stable.

Surveys thus reveal that reading attitudes become more negative across grades, in both elementary and middle school. There has, however, been very little research on changes in attitudes toward reading during the transition between the two, even though reading frequency is known to decline around this time. One cross-sectional study did explore differences in reading attitudes across Grades 1 to 12, but in a very specific population: gifted American students (Anderson et al., 1985). After administering a questionnaire on attitudes toward reading assignments, reading workload, and preference for reading as a leisure time activity, the authors ran analyses of variance (ANOVAs) for four separate groups: primary (Grades 1 to 4), intermediate (Grades 5 and 6), junior high (Grades 7 to 9), and senior high (Grades 10 to 12). Although pupils appeared to maintain a positive attitude toward reading in general, results showed increasingly negative attitudes with age and grade level whatever the reading purpose, with each successive group exhibiting a less positive attitude than the one before. These results suggested that attitude differences across schooling vary little between gifted and unselected students, and that the decline in reading attitudes that begins in elementary school continues into middle or secondary school. Bokhorst-Heng and Pereira (2008) also explored reading attitudes among high achievers, asking 173 pupils in Singapore to complete the Attitudes Toward reading questionnaire at the start and finish of their first year of secondary school. Results indicated a substantial deterioration in positive reading attitudes among pupils during their first year at secondary school. At the end of the year, the pupils perceived themselves to be less proficient readers than they had been at the beginning, and expressed less interest in reading for leisure. By contrast, their attitudes toward reading for learning and academic achievement remained unchanged. To explain these results, the authors hypothesized that the first year of secondary school coincides with the start of adolescence, when increasingly diversified interests potentially compete with reading activities. This could explain the overall worsening of attitudes toward reading during this first year of secondary school. According to Bokhorst-Heng and Pereira (2008), the exam-oriented culture of secondary schools may prove more demanding and stressful than that of elementary schools, partially accounting for the stability of high achievers’ attitudes related to learning, and thus to academic attainment.

In a recent longitudinal study, Kolicì-Vehovec et al. (2014) tracked changes in reading attitudes in unselected pupils aged 10–14 years. A total of 175 Croatian elementary school children completed an adapted version of the ERAS (McKenna and Kear, 1990) three times, in Grades 4, 6, and 8 (it should be noted that in the Croatian education system, the elementary level extends from first to eighth grade). The authors found that positive attitudes toward reading declined significantly between 10 and 14 years, that is, at the start of adolescence. Whereas positive attitudes toward leisure reading deteriorated steadily between Grades 4 and 8, positive attitudes toward academic reading declined steeply between Grades 6 and 8. In the wake of Bokhorst-Heng and Pereira (2008), Kolicì-Vehovec et al. (2014) postulated that this deterioration in reading attitudes among pupils between the ages of 10 and 14 years was partly the result of gaining access to a wider range of leisure activities at adolescence, activities that are often more attractive to this age group than reading. As pointed out by Pitcher et al. (2007), Kolicì-Vehovec et al. (2014) hypothesized that adolescents view reading as an essentially academic activity, and did not count the reading of magazines, newspapers or content on a computer screen as a proper reading activity, thus contributing further to the negative reading attitudes reported in surveys.

### Reading Attitudes by Sex

As shown by a number of studies, most of which were cross-sectional, attitudes toward reading vary significantly according to grade, but also according to sex, with girls expressing more positive views, both in elementary school (McKenna et al., 1995; Sainsbury and Schagen, 2004; Worrel et al., 2006; Logan and Johnston, 2009; Graham et al., 2012; Clark, 2014; McGeown et al., 2015) and in middle school (Swalander and Taube, 2007; McKenna et al., 2012). In a longitudinal study conducted in Germany, from the beginning of kindergarten to the end of first grade, Wolter et al. (2015) reported greater motivation to learn to read in girls as early as kindergarten, owing to the negative impact on boys’ motivation of teachers’ gender role attitudes. In the United States, McKenna et al. (1995) showed that girls’ reading attitudes are consistently more positive than boys’ across Grades 1 to 6 of elementary school. Forshey (2013, Unpublished) reported similar findings, based on a cross-sectional study of 476 elementary-school pupils. In the United Kingdom, both Sainsbury and Schagen (2004) and Logan and Johnston (2009) observed significant sex-related differences in attitudes among pupils in the second half of their elementary education, with girls expressing more positive views, as did McKenna et al. (2012) among American middle-school students. In their longitudinal study, Kolicì-Vehovec et al. (2014) recorded similar results among Croatian pupils, who completed an adapted version of the ERAS (McKenna and Kear, 1990) three times, in Grades 4, 6, and 8 (i.e., second half of elementary school in the Croatian system), with girls’ reading attitudes being consistently more positive than boys’ across those grades.

Exactly how these sex-related differences in reading attitudes change across the elementary and middle/secondary levels seems to depend on the reading goal, although results have so far been contradictory. McKenna et al. (1995), for instance, found that girls’ and boys’ attitudes toward academic reading changed to the same extent during elementary school, with these attitudes becoming less positive with each successive academic year. In a longitudinal study, Kush and Watkins (1996) undertook a survey of 190 pupils in Grades 1 to 4. They failed to find any difference between girls and boys in their attitudes toward academic reading, while an initial gap in terms of leisure reading persisted across grades.

### The Present Study

In sum, the various studies undertaken to describe and understand changes in pupils’ reading attitudes have consistently reported (i) a worsening of positive attitudes toward reading with age and grade level, across both the elementary and middle/secondary levels (McKenna et al., 1995, 2012; Hogsten and Peregoy, 1999; Lazarus and Callahan, 2000; Kolicì-Vehovec et al., 2014), (ii) better attitudes toward leisure reading than toward academic reading (Ley et al., 1994; McKenna et al., 1995, 2012), (iii) more positive attitudes toward leisure and academic reading in girls than in boys (McKenna et al., 1995, 2012; Sainsbury and Schagen, 2004; Worrel et al., 2006; Swalander and Taube, 2007; Logan and Johnston, 2009; Graham et al., 2012; Clark, 2014; McGeown et al., 2015), and (iv) a deterioration in positive attitudes toward reading across grades, with an earlier and sharper decline among boys than among girls, in the case of leisure reading (McKenna et al., 1995). This decline does not differ according to sex for academic reading (McKenna et al., 2012).

These results raise several questions.

First, although there appears to be an earlier and sharper decline for academic reading, current data are inconclusive when it comes to the precise nature of this decline, especially beyond elementary school. Surveys conducted among middle-school pupils have been mainly American. They show that attitudes toward academic reading remain stable, albeit not particularly positive, between Grades 6 and 8. One explanation for these results is that when pupils move from elementary to middle school, they have to contend with more demanding and stressful curricula, and therefore come to associate reading for learning with academic achievement. In other countries, however, some studies, notably Kolicì-Vehovec et al. (2014)’s survey of Croatian pupils aged 10–14 years, have reported a sudden worsening of positive attitudes toward academic reading between Grades 6 and 8. In Croatia, elementary school lasts 8 years (i.e., until age 14 years). The sudden worsening of positive attitudes toward academic reading observed between Grades 6 and 8 does not, therefore, coincide with the move into secondary school. The results of Kolicì-Vehovec et al. (2014)’s study therefore support the hypothesis that attitudes toward reading deteriorate at the start of adolescence, when young people’s interests begin to diversify and they gain access to a broader range of leisure activities which then compete with reading (Johnsson-Smaragdi and Jönsson, 2006).

Second, the transition from elementary to middle or secondary school has been particularly poorly documented. This period, which generally also marks the start of adolescence, coincides with a major change in reading habits (Johnsson-Smaragdi and Jönsson, 2006). Nevertheless, there has been little research on changes in attitudes toward reading during the elementary–middle/secondary transition, with most current data coming from surveys of high achievers or gifted students (Anderson et al., 1985; Bokhorst-Heng and Pereira, 2008), rather than from unselected readers, and although some results point to a similar attitude change in both populations, we cannot assume this to be the case. Moreover, although sex-related differences (i.e., more positive attitudes toward both leisure and academic reading among girls) have also been observed from the start of elementary school, continuing into middle school, little is known as to why these differences occur with grade, according to reading goals (leisure vs. academic).

Third, despite the diversity of the pupil populations included in these surveys, in terms of age, grade level, geographical origin and learner profile, the vast majority of studies have been conducted among English-speaking pupils in North America. For this reason, it is important to undertake research among French speakers in North America, in order to document different language-related practices but within a similar, North American education system, rather than a European one featuring a different curriculum.

The present study was therefore specifically designed to respond to these questions by describing differences in reading attitudes–according to sex and reading goal–among young French-speaking readers in Quebec (Canada) between the end of elementary school and the beginning of middle/secondary school. As the transition between elementary and middle/secondary schools takes place between Grades 7 and 8 (i.e., at age 12 years) in Quebec, we set out to describe differences between Grades 5 and 8 (i.e., encompassing the last two grades of elementary school and the first two grades of secondary school), in order to assess whether the expected decrease in reading attitudes between the two levels is linear (gradual) or non-linear (sudden).

Moreover, in order to adequately and consistently complement previous studies with findings for the French language and Canadian context, we chose to use the survey developed by McKenna and Kear (1990) and McKenna et al. (2012), which has already yielded a great deal of data, adapting it to the context of our study.

Based on previous studies, we expected to confirm the following hypotheses about the main and interaction effects of grade, type of reading, and sex:

(i) Whatever their sex or the type of reading, pupils in middle school (Grades 7 and 8) have less positive attitudes toward reading than pupils in elementary school (Grades 5 and 6). Moreover, the greatest difference in attitudes is between Grades 6 and 7 (i.e., transition from elementary to middle school);
(ii) Whatever their grade and sex, students have more positive attitudes toward leisure reading than toward academic reading. Nevertheless, the superiority of leisure reading decreases with grade;
(iii) Whatever their grade and the type of reading, girls have more positive attitudes toward reading than boys, particularly for leisure reading;
(iv) The effect of grade (i.e., decrease in positive attitudes, especially between Grades 6 and 7) is greater for boys than for girls, especially in the case of leisure reading.

## Materials and Methods

In this section, we describe our sample, the survey items and rating scale, and the procedure for administering the survey.

### Participants and Setting

Participants were recruited using a non-probabilistic sampling method. The Research Chair in Reading and Writing Learning in Young Children (CREALEC) facilitated the identification of participant schools through its partnerships with various school boards and schools in Quebec. The study was carried out in accordance with the ethical guidelines for research established by Sherbrooke University’s education and social sciences ethics committee. All parents were asked to provide their written informed consent prior to data collection. We only included children whose parents had given this consent. Parents and students were informed that participation was voluntary, and they could opt out at any time.

As shown in Table 1, a total of 468 children (233 girls and 235 boys), including 201 drawn from seven elementary schools and 267 drawn from three middle schools, took part in the study. Table 1 shows the composition of the sample according to sex and grade level.

**Table 1:** Breakdown of sample according to sex and grade level.

		*n* Girls	*n* Boys	*n* Total
Elementary school	Grade 5	50	60	110
	Grade 6	43	48	91
Subtotal		93	108	201
Middle school	Grade 7	108	56	164
	Grade 8	32	71	103
Subtotal		140	127	267
Total		233	235	468


As a group, participants were predominantly French-speaking students from Quebec (Canada), reflecting the school boards from which they came. Low-income and socio-economic deprivation indices were considered in the selection of participant schools (Ministry of Education, Recreation and Sports, 2011). Based on these two indices, Quebec schools are ranked on a scale of 1 (least disadvantaged) to 10 (most disadvantaged), and data on the students’ backgrounds (by school) showed a relatively balanced distribution of the sample as a whole, between disadvantaged, average and advantaged backgrounds.

Table 2 shows the low-income (and decile rank) and socio-economic deprivation (and decile rank) indices of each participating school. The low-income cut-off is defined as the level at which families are estimated to spend 20% more than the overall average on food, shelter and clothing, in terms of the proportion of their total income. It provides a means of estimating the proportion of families whose income may be considered low, taking into account the size of the family and the place of residence (rural area, small urban area, large metropolitan area) (Government of Quebec, 2018). The socio-economic index can be used to establish the proportion of families with children where the mother does not have any diploma, certificate or degree (two-thirds of the index weighting) and the proportion of households where the parents were not in employment at the time of the Canadian census of reference (one-third of the index weighting) (Government of Quebec, 2018).

**Table 2:** Breakdown of sample according to grade level, sex, and low-income, and deprivation indices.

School	Grade	*n* Girls	*n* Boys	*n* Total	Low income index		Socio-economic deprivation index	
**Elementary schools**					
1	5	8	15	23	12.07	5	10.24	5
2	5	1	6	7	11.94	5	15.16	8
	6	2	4	6				
3	5	21	18	39	7.54	2	8.66	4
	6	8	9	17				
4	5	5	5	10	11.80	5	10.96	6
	6	22	14	36				
5	5	15	16	31	12.18	5	5.95	2
	6	11	21	32				
**Middle schools**					
6	7	52	44	97	9.77	2	12.42	6
	8	14	11	25				
7	8	17	61	78	12.11	5	12.23	5
8	7	56	11	67	29.62	9	14.95	8
	8	1	0	1				


Schools and teachers participated on an entirely voluntary basis. All the pupils who agreed to take part were included in the data collection. Participants came from mainstream classes and schools. According to their schools, they had no developmental, behavioral, or cognitive problems or delay.

### Survey Instrument

For the purposes of the survey, we administered an adapted version of the ERAS (McKenna and Kear, 1990; McKenna et al., 1995) to measure the constructs of recreational and academic reading attitudes.

#### Item Development

Before selecting the instrument’s items, we considered past and recent studies of academic and recreational reading, and examined surveys addressing attitudes toward these two types of literacy in elementary and middle-school students. It should be noted that in the present study, we chose not to include items that referred to reading in digital settings, unlike McKenna et al. (2012) in their survey examining the current state of reading attitudes among middle-school students. There were two reasons for this choice. First, recent studies in Quebec on the introduction of technological tools in the classroom (e.g., Fieìvez and Karsenti, 2018) tend to show that teachers use new technologies in much the same way that they use texts in print settings (e.g., accessing and sharing information). Second, as far as leisure reading in digital settings is concerned, we did not accept the very broad conception of digital literacy adopted in some previous studies, such as being on social websites during free time and reading e-mails from friends (see McKenna et al., 2012), as we did not consider these particular activities to come under the heading of digital literacy (see Martin, 2008).

As McKenna and Kear (1990)’s questionnaire seemed representative of what we wanted to capture in terms of attitudes toward reading in the target population, we used it as a working list of possible items. This list was then submitted to two consultants involved in the Action Plan on Reading in School, a reading program supported by the Youth Training Department of Quebec’s Ministry of Education and Higher Education^2^. These consultants served as experts, drawing on their extensive knowledge of the current reading practices of students in Quebec school boards to determine whether the items in the list targeted reading practices associated with one of the two constructs. By this means, we refined and narrowed the list of items to eliminate what seemed to be redundancies in each subscale. For example, the items “How do you feel when you read a book on a rainy Saturday?” “How do you feel about reading a book in your free time?” and “How do you feel about spending free time reading?” were judged to be similar, and thus redundant, by the experts (see Table 3).

**Table 3:** Dimensions and items adapted from McKenna and Kear (1990)’s survey instrument.

McKenna and Kear (1990)’ instruments items	Items retained	Items removed
**Leisure reading**		
1. How do you feel when you read a book on a rainy Saturday?		Items 1, 3, 5, and 8 judged similar. Item 3 was retained
2. How do you feel when you read a book in school during free time?	x	
3. How do you feel about reading for fun at home?	x	
4. How do you feel about getting a book for a present?	x	
5. How do you feel about spending free time reading?		Items 1, 3, 5, and 8 judged similar. Item 3 was retained
6. How do you feel about starting a new book?	x	
7. How do you feel about reading during summer vacation?	x	
8. How do you feel about reading instead of playing?		Items 1, 3, 5, and 8 judged similar. Item 3 was retained
9. How do you feel about going to a bookstore?		Items 6, 9, and 10 judged similar. Item 6 was retained
10. How do you feel about reading different kinds of books?		Items 6, 9, and 10 judged similar. Item 6 was retained
**Academic reading**		
11. How do you feel when the teacher asks you questions about what you read?	x	
12. How do you feel about doing reading workbook pages and worksheets?		Items 12, 13, 16, and 17 were judged similar. Item 16 was retained.
13. How do you feel about reading in school?		Items 12, 13, 16, and 17 were judged similar. Item 16 was retained.
14. How do you feel about reading your school books?		Items 11, 14, and 19 were judged similar. Item 11 was retained.
15. How do you feel about learning from a book?	x	
16. How do you feel when it’s time for reading class?	x	
17. How do you feel about the stories you read in reading class?		Items 12, 13, 16, and 17 were judged similar. Item 16 was retained.
18. How do you feel when you read out loud in class?	x	
19. How do you feel about using a dictionary		Items 11, 14, and 19 were judged similar. Item 11 was retained.
20. How do you feel about taking a reading test?	x	


Based on these judgments, 10 items were retained, half probing leisure reading and half academic reading (McKenna and Kear, 1990; McKenna et al., 1995). As with McKenna and Kear (1990)’s original instrument, participants in our survey were asked to rate each item on a simple four-point scale ranging from 1 (*I don’t like it*) to 4 (*I love it*), as some of our participants were in elementary school. Research has suggested that younger children have difficulty discriminating between more than five information units at the same time (McKenna et al., 1995). Moreover, the even number of points had the added benefit of avoiding a central category, thus forcing participants to commit themselves (Henk et al., 2011). As our study was aimed at Grades 5 to 8, we chose not to use the pictorial rating format favored by McKenna and Kear (1990) for use with elementary-school children. We calculated a mean score for each dimension of the questionnaire (i.e., leisure reading and academic reading). To assess the factor structure of this abridged version of the questionnaire, we conducted a principal component analysis with SPSS 23 software, based on the 10 items and using an oblique promax rotation (justified by a high factor correlation: *r* = 0.56). Results showed a significant effect of model (χ^2^ = 228, *df* = 26, *p* < 0.0001), and the presence of two factors corresponding to leisure and academic reading (see Table 4).

**Table 4:** Principal component analysis, item loadings, and final selection of items that contributed most to the questionnaire’s factor structure (loading on corresponding factor > 0.70).

Reading	Item	Factor 1	Factor 2	Item retained
Pleasure	Voici comment je me sens quand je lis pendant mes temps libres à l’école (How do you feel when you read a book in school during free time?)	0.652	0.231	
	Voici comment je me sens quand je lis pendant mes temps libres à la maison (How do you feel about reading for fun at home?)	0.893	–0.070	x
	Voici comment je me sens quand je reçois un livre en cadeau (How do you feel about getting a book for a present?)	0.897	–0.101	x
	Voici comment je me sens quand je commence la lecture d’un nouveau livre (How do you feel about starting a new book?)	0.838	0.040	x
	Voici comment je me sens quand je lis pendant mes vacances d’été ou mes jours de congé (How do you feel about reading during summer vacation?)	0.911	–0.153	x
Academic	Voici comment je me sens quand mon enseignant me pose des questions sur ce que j’ai lu en classe (How do you feel when the teacher asks you questions about what you read?)	–0.022	0.823	x
	Voici comment je me sens quand je dois lire à haute voix en classe (How do you feel when you read out loud in class?)	–0.231	0.855	x
	Voici comment je me sens quand c’est le temps de lire en classe (How do you feel when it’s time for reading class?)	0.547	0.379	
	Voici comment je me sens quand j’apprends des choses dans un livre à l’école (How do you feel about learning from a book?)	0.282	0.527	
	Voici comment je me sens quand je commence la lecture d’un nouveau livre (How do you feel about starting a new book?)	–0.029	0.747	x


To enhance the power of the scale, we selected items that were coherently linked to the factors, with loadings of at least 0.70. Based on these criteria, we retained seven items to efficiently evaluate differences in attitudes toward reading in students: four for leisure reading, and three for academic reading. The reliability of the constructs, as assessed by Cronbach’s alpha coefficient of internal consistency, was good (α = 0.83; varying from 0.786 to 0.842 when items were dropped).

### Survey Administration

Data were collected over two consecutive academic years (2012–2013 and 2013–2014), between the months of February and May, from different schools and sets of students (i.e., cross-sectionally). Before collecting these data, the participating research assistants met with the principal researcher for a 3-h training session, and were given a data collection protocol to follow, to ensure that the procedure was rigorously implemented. We met the pupils as a group, in the school’s IT department, as the survey was computer-based and had to be accessed via the web. Two or three experimenters supervised the pupils while they responded to the survey. Verbal instructions were given at the start, after which the pupils responded to the survey on their own and at their own pace (10 min was enough to complete the survey). The experimenters took care to answer every question and clarify the instructions, if requested or required by pupils during the session. To avoid a social desirability bias, teachers were asked to stay out of the room while their pupils completed the questionnaire. As and when the pupils finished, they returned to their classroom.

## Results

Results of the survey are presented in Table 5. To analyze changes in pupils’ attitudes toward reading according to grade level, sex and type of reading, we ran three-factor (Grade × Type of reading × Sex) ANOVAs with SPSS 23 software. We examined the interaction effects by means of partial comparisons, when subtended by a hypothesis, and by *post hoc* (Bonferroni) multiple comparisons when we needed to examine a particular effect.

**Table 5:** Means (and standard deviation) of attitudes toward leisure and academic reading among boys and girls across Grades 5 and 6 (elementary school) and 7 and 8 (middle school).

		Girls		Boys	
		Leisure reading	Academic reading	Leisure reading	Academic reading
Elementary	Grade 5	3.25 (0.61)	2.41 (0.73)	2.76 (0.71)	2.09 (0.72)
	Grade 6	3.07 (0.72)	2.35 (0.55)	2.86 (0.77)	2.34 (0.68)
Middle	Grade 7	2.73 (0.89)	1.89 (0.69)	2.03 (0.83)	1.79 (0.67)
	Grade 8	2.32 (0.97)	1.71 (0.63)	1.80 (0.70)	1.89 (0.68)


An ANOVA showed that the main effect of grade was significant, *F*(3,460) = 37.22, *MS* = 27.01, *p* < 0.001, η^2^ = 0.187. However, a contrast analysis (pairwise, Bonferroni-adjusted comparison) failed to reveal any significant difference in attitude scores between fifth graders (*M* = 2.61) and sixth graders (*M* = 2.65). By contrast, attitude scores fell significantly between Grades 6 (i.e., final year of elementary school) and 7 (i.e., first year of middle school; *M* = 2.17, *p* < 0.001). There was no significant difference between Grades 7 and 8 (*M* = 1.90).

The main effect of type of reading was significant, *F*(1,460) = 176.93, *MS* = 62.07, *p* < 0.001, η^2^ = 0.257, with attitude toward leisure reading (*M* = 2.58) scoring higher than attitude toward academic reading (*M* = 2.04).

The main effect of sex was also significant, *F*(1,460) = 17.63, *MS* = 15.35, *p* < 0.001, η^2^ = 0.035, with boys (*M* = 2.16) having less positive attitudes than girls (*M* = 2.45).

Regarding interactions between the factors, the effect of the Grade × Type of reading interaction was significant, *F*(3,460) = 5.95, *MS* = 2.09, *p* < 0.001, η^2^ = 0.026. *Post hoc* comparisons indicated that the superiority of leisure reading over academic reading gradually diminished across the four grades, such that the difference was no longer significant by Grade 8 (+0.75, *p* < 0.001; +0.62, *p* < 0.001; +0.63, *p* < 0.001; +0.13, *p* > 0.15). The Grade × Sex interaction was not significant. The effect of the Sex × Type of reading interaction was significant, *F*(1,460) = 26.02, *MS* = 9.12, *p* < 0.001, η^2^ = 0.038. The attitude score was higher for leisure reading than for academic reading, especially among girls (+0.79, *p* < 0.001 for girls vs. +0.31, *p* < 0.001 for boys). Lastly, the effect of the second-order interaction between the three factors was significant, *F*(3,460) = 2.65, *MS* = 0.93, *p* < 0.05, η^2^ = 0.012. The superiority of leisure reading over academic reading persisted among girls across the grades (*p* < 0.002), whereas it was no longer significatively present among boys in Grades 7 and 8 (*p* > 0.006 after Bonferroni adjustment, with *p* < 0.001 in Grades 5 and 6).

## Discussion

The present study was designed to examine differences in reading attitudes among young readers between the end of elementary school and the beginning of middle school, according to sex and type of reading. Based on previous findings, we expected to observe differences in attitudes toward reading in participants during this transitional period (i.e., from Grades 5 and 6 to Grades 7 and 8), with pupils in elementary school (Grades 5 and 6) having more positive attitudes toward reading, whatever their sex or the type of reading, than pupils in middle school (Grades 7 and 8). Moreover, this difference in attitudes would concern the transition between elementary and middle schools (i.e., between Grades 6 and 7).

We also expected to see more marked differences in attitudes toward academic reading as opposed to leisure reading, and a widening gap between boys and girls (favoring girls), based on previous findings.

As expected, results showed differences in pupils’ attitudes between Grades 6 and 7, corresponding to the transition from elementary to middle school for our participants. Sixth graders exhibited more positive attitudes toward reading than seventh graders. However, we failed to find significant differences either between Grades 5 and 6 (i.e., last 2 years of elementary school) or between Grades 7 and 8 (i.e., first 2 years of middle school). Anderson et al. (1985) had noted a significant worsening of reading attitudes between these two cycles among gifted students, but whereas these authors observed a steady decline in attitudes across elementary and secondary education, our results indicated that the only significant difference in attitudes toward reading was between the final year of elementary school (Grade 6) and the first year of middle school (Grade 7). Our finding of relative stability in attitudes toward reading during the last 2 years of elementary school and then during the first 2 years of middle school contrasts with previous findings, both for elementary school (McKenna et al., 1995; Hogsten and Peregoy, 1999; Lazarus and Callahan, 2000) and for middle school (Ley et al., 1994). Instead, it tends to confirm the results of Petscher (2010)’s meta-analysis, which highlighted a smaller grade effect among elementary school children than among middle-school students. When McKenna et al. (2012) conducted their study in middle school, they too found only weak grade-related variance, even if there was a “slight, non-monotonic worsening” of attitudes toward reading across middle school.

At first sight, the difference in reading attitudes we observed between the end of elementary school (Grades 5 and 6) and the start of middle school (Grades 7 and 8), with attitudes growing more negative, suggests that the transition between the two levels should be regarded as one of the factors influencing young people’s attitudes toward reading. Johnsson-Smaragdi and Jönsson (2006) emphasized that “the age between 9 and 12 is usually considered as ‘the book devouring age,’ whereafter the interest in reading tends to decrease” (p. 522). This could partially account for the negative trend observed among the pupils in our sample as they entered middle school, but we are left with the question as to why this book-devouring phase peters out at 12 years. Only a more detailed analysis of the links between this developmental change and other factors will allow us to formulate explanatory hypotheses. Research conducted with pupils in the elementary or middle/secondary level (Gervais, 2000/2001; Hooper, 2005; Johnsson-Smaragdi and Jönsson, 2006) has shown that young readers tend to read less and less as they go through school. Reading habits may depend on a number of factors, including reading environment (Keskin and Bastug, 2014; Sénéchal and LeFevre, 2014; Hume et al., 2015). For example, a rich reading environment both at home and in preschool (including the availability of interesting books, but also–in the case of boys–a teacher fostering less gender-typed attitudes and behaviors conveying the idea that reading is a feminine activity) is associated with more positive attitudes toward or interest in reading among young children (Cunningham, 2008; Hume et al., 2015; Wolter et al., 2015). Similarly, studies conducted with secondary-school pupils have revealed links between the richness of the reading environment at home, the reading habits (in terms of frequency) of family members, including the students themselves, and the latter’s attitudes toward reading (Keskin and Bastug, 2014).

Regarding changes in attitude according to type of reading (Grade × Type of reading interaction), in our study among pupils from Grades 5 to 8, we found more positive attitudes toward leisure reading than toward academic reading, even though there was a negative trend in attitudes toward both types of reading across grades, in line with previous studies among elementary or middle/secondary school pupils (McKenna et al., 1995, 2012). As we had predicted, differences in attitudes were more marked for academic reading than for leisure reading across grades. However, the gap between attitudes toward leisure versus academic reading gradually narrowed across grades, such that it had ceased to be significant by Grade 8. These results can be interpreted in the light of Bokhorst-Heng and Pereira (2008)’s findings. These authors reported a decline in high achievers’ attitudes toward leisure reading in their first year of secondary school, whereas their attitudes toward academic reading, more closely associated with learning and thus with academic success, remained stable. In our study, where the pupils were unselected, attitudes toward leisure reading continued to decline in middle school, but less steeply than before.

It is also possible that the results of our survey only concern differences in attitudes toward reading in the traditional medium (i.e., print), as none of the items in our survey explicitly referred to reading in digital settings. Pitcher et al. (2007) and McKenna et al. (2012) showed that attitudes can be expected to vary, not only according to the perceived purpose–academic or leisure–of the literacy activity, but also according to the medium in which this activity takes place. Hutchinson and Henry (2010) reported differences in the use of and preferences for digital activities between preadolescents (Grades 4 and 5) and adolescents (Grades 6 to 8). Even though the former said they preferred to use the Internet rather than print for reading activities, they believed it was harder to use the Internet for reading than a book. They also believed they would learn more from a book than from the Internet. By contrast, adolescents seem to have extensive digital literacy habits out of school (Alvermann, 2008).

Nonetheless, McKenna et al. (2012)’s survey showed that attitudes toward academic reading do not vary across media (i.e., print vs. digital). These authors postulated that this result reflected the tendency of middle-school teachers to incorporate technology into teaching in much the same way as they do printed texts. They argued that, as a result, the potential of digital environments to increase students’ engagement remains untapped, and attitudes toward reading remain similar across these two settings. Future research should address this issue, in the light of findings from previous studies such as theirs. McKenna et al. (2012)’s survey also showed that adolescents tend to prefer recreational reading activities in digital settings, but here again, no outright differences emerged in terms of attitudes toward specific reading activities in print versus digital settings. It is worth noting that all the recreational digital items in their questionnaire involved reading texts produced by peers or friends. However, many educators and researchers question McKenna et al. (2012)’s premise that the activities that adolescents particularly enjoy in digital environments, such as viewing Facebook pages or what a friend has posted on social media, actually constitute recreational reading activities.

Regarding differences between boys’ and girls’ reading attitudes, our data showed a persistent sex effect from fifth to eighth grade. Overall, these results corroborate those of previous research (McKenna et al., 1995, 2012; Worrel et al., 2006; Swalander and Taube, 2007; Logan and Johnston, 2009) showing a sex effect throughout schooling. In the present study, girls’ attitudes between fifth and eighth grade proved more positive than boys’, for both leisure and academic reading. The second-order Grade × Type of reading × Sex interaction was also significant, showing that the superiority of leisure reading over academic reading persisted among girls across the grades, whereas it was no longer significatively present among boys in Grades 7 and 8. Thus, we can conclude that, as far as our study is concerned, goal-related reading attitudes differed according to sex between Grades 5 and 8. However, our study design did not allow us to explain the sex-related difference in attitudes toward reading. Future research should therefore address this issue.

As our study was intended to describe differences in attitudes toward reading during the transition from elementary to middle school, we did not consider links between these attitudes and actual reading performances, even though this “complex and possibly reciprocal” (McKenna et al., 2012, p. 287) relationship is well documented (Petscher, 2010). Nor did our study design allow us to explain the sex-related difference in attitudes toward reading. Poorer reading performances among boys could not account for McKenna et al. (1995)’s results, as the boys and girls in their sample had comparable reading skills. Both McKenna et al. (1995) and Johnsson-Smaragdi and Jönsson (2006) have suggested that the sex gap can instead be explained by cultural expectations, which are higher for girls in this respect. Wolter et al. (2015) also emphasized the effect of kindergarten teachers’ reading-related gender stereotypes, whereby reading is for girls, in lowering boys’ motivation for reading.

This hypothesis that the sex gap can be explained by cultural expectations and gender stereotypes is supported by the results of the longitudinal study by Archambault et al. (2010), which indicated that boys place less subjective value on reading than girls throughout the whole of their elementary and secondary schooling.

Lastly, despite the diversity of the pupil populations included in previous surveys, the vast majority of research has so far been conducted among English-speaking pupils in North America. Our study was therefore intended to describe differences in attitudes toward reading in North America, but among French-speaking pupils. This allowed us to document attitudes toward reading, focusing on the transition from elementary to middle school, in a context with different linguistic practices but a similar education system, rather than a European one featuring a different curriculum. Overall, our results corroborate those of previous research, regarding differences in reading attitudes according to sex and type of reading (leisure vs. academic), showing a persistent sex effect among pupils in Grades 5 to 8, favoring girls (McKenna et al., 1995, 2012), and more positive attitudes toward leisure reading than toward academic reading, even though there was a negative trend in attitudes toward both types of reading across grades (Ibid.). However, our finding that significant differences in reading attitudes were restricted to the period of transition from elementary to secondary school contrasts with previous findings, which generally show a steady decline in attitudes across both elementary school (McKenna et al., 1995; Hogsten and Peregoy, 1999; Lazarus and Callahan, 2000) and middle school (Ley et al., 1994). The how and why of these changes during the move from elementary to secondary school in our study context are still unclear, but the transition between the two levels should probably be regarded as one of the factors influencing young people’s attitudes toward reading, at least in some contexts. These discrepant results also suggest that we cannot assume that the results of research on this subject are transferable, thus underlining the relevance of closely documenting these differences and changes in attitudes toward reading among pupils in the course of their schooling in a range of linguistic and education contexts.

### Limitations and Implications for Future Research

The present study had several limitations. First, our survey took the form of a self-report questionnaire, meaning that our results should therefore be viewed with caution, owing to the social desirability bias associated with this type of instrument. Furthermore, using a Likert-like rating scale to measure attitudes means relying on respondents’ subjective judgments. In addition, as our sample was made up of volunteers, it was not randomized, and mostly consisted of French-speaking Canadian students. Our results cannot, therefore, necessarily be considered as being representative of the reality of certain multi-ethnic school environments. That being said, all socioeconomic backgrounds were relatively equally represented in our sample.

Moreover, as noted above, our survey did not take account of attitudes toward reading in different media (i.e., reading in both traditional and digital settings). Future research should explore non-traditional media, as this could provide a clearer picture of pupils’ attitudes toward reading during the transition from elementary to middle school.

In addition, as ours was a cross-sectional study, we could not track individual participants’ reading attitudes over time. Longitudinal studies are therefore needed to provide a more finely grained picture of changes in attitudes toward leisure and academic reading across schooling, especially during the transition from elementary to middle/secondary education.

Also, our results did not allow us to explain the differences in pupils’ attitudes toward reading, especially the significant negative trend at the start of middle school. We can only make assumptions, based on previous research highlighting the many changes that take place, both socially and academically, during the transition from elementary to middle school, as well as the disturbances associated with the onset of adolescence.

Future research should therefore examine changes in the reading habits of young readers between Grades 1 and 11 as well as any modifications to their reading environment during this period. The links between pupils’ reading habits, reading environment and attitudes toward reading should also be explored across education levels, in order to identify the factors behind the changes observed in reading attitudes, especially between the end of elementary school and the beginning of middle school, a period that seems to be characterized by significant changes in both reading attitudes and reading habits. This would also shed light on the sex gap in reading attitudes and, consequently, in reading habits across schooling.

The present survey should also be complemented by studies that distinguish between different reader profiles in elementary and middle school, as this would help to explain developmental differences in attitudes toward reading. In particular, case studies featuring *in situ* observations and interviews could build on our results by exploring some of the socioaffective and cognitive factors that contribute not only to reading skills –as emphasized in earlier studies (e.g., Jenkins et al., 2003; Gillioz et al., 2012; Unsworth and McMillan, 2013)– but also to reading attitudes among young readers. Such studies would provide an opportunity to assess how individual differences shape the profiles of young readers, with a view to tailoring reading instruction to their diverse cognitive and socioaffective needs throughout schooling.

## Conclusion

We conducted the present investigation to extend previous research by examining reading attitudes among pupils as they move from elementary school to middle school. Results showed that attitudes toward reading in general (i.e., regardless of reading goal) remained stable among participants, except during the elementary–middle school transition, when we observed a significant difference in pupils’ attitudes, which grow more negative during this period of schooling. Our results do not, therefore, corroborate previous reports of a broadly negative trend in students’ reading attitudes across education levels.

Regarding attitude differences in relation to reading goal, as in several previous studies, we found that attitudes toward leisure reading remained more positive than attitudes toward academic reading, at least until Grade 8, when there ceased to be any significant difference between the two.

As for attitude differences according to sex, our data showed that girls’ attitudes were more positive than boys’ toward both leisure and academic reading. Overall, our results corroborate previous findings of a sex effect throughout schooling.

The value of our investigation stems partly from the fact that it was designed to focus on differences in reading attitudes during the elementary–middle school transition which, to our knowledge, had not previously been investigated –at least not among typically developing pupils. In addition, there is scant relevant research on this topic among young readers in Quebec (Canada). Our research highlighted a general negative trend of students’ attitudes toward reading between Grades 6 and 7, thereby helping to build up a more detailed picture of attitude change between the beginning and end of schooling in Quebec.

## Ethics Statement

This study was carried out in accordance with the recommendations of Sherbrooke University’s education and social sciences ethics committee. The parents/guardians of all the participants gave their written informed consent, in accordance with the Declaration of Helsinki. The protocol was approved by Sherbrooke University’s education and social sciences ethics committee.

## Author Contributions

All the authors agreed to be accountable for the content of the work. They all collaborated in the planning, data analysis, and writing stages.

## Conflict of Interest Statement

The authors declare that the research was conducted in the absence of any commercial or financial relationships that could be construed as a potential conflict of interest.

## Footnotes

1 In this survey, Grade 6 corresponded to the first year of middle school.

2 “The Reading at School Action Plan is intended to create practices that will allow young people to enjoy reading, make regular and effective use of reading, and make reading a lifelong habit. It is aimed at preschool, elementary and high-school students, particularly boys, as well as school staff who work with youth on reading. Parents are also targeted, so that they are made aware of the importance of reading” (Government of Quebec, 2018, p. 5).

## References

[B1] Ajzen I. Fishbein M. (2005). “The influence of attitudes on behavior,” in *The Handbook of Attitudes*, eds Albarracin D. Johnson B. T. Zanna M. P. (Mahwah, NJ: Erlbaum), 173–221.

[B2] Alexander P. A. Fox E. (2011). “Adolescents as Readers,” in *Handbook of Reading Research*, eds Kamil M. L. Pearson P. D. Moje E. B. Afflerbach P. P. (New York, NY: Routledge), 157–176.

[B3] Alvermann D. E. (2008). Why bother theorizing adolescents’ online literacies for classroom practice and research? *J. Adolesc. Adult Lit.* 52 8–19. 10.1598/JAAL.52.1.2

[B4] Anderson M. A. Tollefson N. A. Gilbert E. C. (1985). Giftedness and reading: a cross-sectional view of differences in reading attitudes and behaviors. *Gift. Child Q.* 29 186–189. 10.1177/001698628502900411

[B5] Anmarkrud O. Braten I. (2009). Motivation for reading comprehension. *Learn. Individ. Differ.* 19 252–256. 10.1016/j.lindif.2008.09.002

[B6] Archambault I. Eccles J. S. Vida N. (2010). Ability self-concepts and subjective value in literacy: joint trajectories from grades 1 through 12. *J. Educ. Psychol.* 102 804–816. 10.1037/a0021075

[B7] Baker L. Wigfield A. (1999). Dimensions of children’s motivation for reading and their relations to reading activity and reading achievement. *Read. Res. Q.* 34 452–477. 10.1598/RRQ.34.4.4

[B8] Becker M. McElvany N. Kortenbruck M. (2010). Intrinsic and extrinsic reading motivation as predictors of reading literacy: a longitudinal study. *J. Educ. Psychol.* 102 773–785. 10.1037/a0020084

[B9] Benner A. D. (2011). The transition to high school: current knowledge, future directions. *Educ. Psychol. Rev.* 23 299–328. 10.1007/s10648-011-9152-0 21966178 PMC3182155

[B10] Bokhorst-Heng W. Pereira D. (2008). Non-at-risk adolescents’ attitudes towards reading in a Singapore secondary school. *J. Res. Read.* 31 285–301. 10.1111/j.1467-9817.2008.00369.x

[B11] Chouinard R. Bowen F. Fallu J.-S. Lefrançois P. Poirier L. (2015). *La Transition au Secondaire et l’Incidence des Mesures de Soutien sur la Motivation, L’Adaptation Psychosociale et les Apprentissages des Élèves (Rapport de Recherche, Programme de Recherche sur la Persévérance et la Réussite Scolaires, Action Concertée FQRSC-MELS).* Montreal: University of Montreal.

[B12] Clark C. (2014). *The Reading Lives of 8 to 11-Year-Olds 2005–2013 An Evidence Paper for the Read On Get On Coalition.* London: National Literacy Trust.

[B13] Clark C. De Zoysa S. (2011). *Mapping the Interrelationships of Reading Enjoyment, Attitudes, Behaviour and Attainment.* London: National Literacy Trust.

[B14] Conlon E. G. Zimmer-Gembeck M. J. Creed P. A. Tucker M. (2006). Family history, self-perceptions, attitudes and cognitive abilities are associated with early adolescent reading skills. *J. Res. Read.* 29 11–32. 10.1111/j.1467-9817.2006.00290.x

[B15] Conradi K. Jang B. G. Bryant C. Craft A. McKenna M. C. (2013). Measuring adolescents’ attitudes toward reading: a classroom survey. *J. Adolesc. Adult Lit.* 56 565–576. 10.1002/JAAL.183

[B16] Cunningham D. D. (2008). Literacy environment quality in preschool and children’s attitudes toward reading and writing. *Lit. Teach. Learn.* 12 19–36.

[B17] De Naeghel J. Van Keer H. Vansteenkiste M. Rosseel Y. (2012). Relation between elementary students’ recreational and academic reading motivation, reading frequency, engagement, and comprehension: a self-determination theory perspective. *J. Educ. Psychol.* 104 1006–1021. 10.1037/a0027800

[B18] Fieìvez A. Karsenti T. (2018). Usages et perceptions des enseignants lors de l’utilisation de la tablette en contexte scolaire. *Formation Prof.* 26 55–73. 10.18162/fp.2018.394

[B19] Fishbein M. Ajzen I. (1975). *Belief, Attitude, Intention, and Behavior: An Introduction to Theory and Research.* Reading, MA: Addison-Wesley.

[B20] Gambrell L. B. Palmer B. M. Codling R. M. Mazzoni S. A. (1996). Assessing motivation to read. *Read. Teach.* 49 518–533. 10.1598/RT.49.7.2

[B21] Gervais F. (2000/2001). Habitudes de lecture chez les 9-12 ans: une enquête aux résultats qui nous laissent songeurs. *Can. Child. Lit.* 100–101,94–107.

[B22] Gillioz C. Gygax P. Tapiero I. (2012). Individual differences and emotional inferences during reading comprehension. *Can. J. Exp. Psychol.* 66 239–250. 10.1037/a0028625 22774802

[B23] Government of Quebec (2018). *Action Plan on Reading in School.* Available at: http://www.education.gouv.qc.ca/dossiers-thematiques/lecture/

[B24] Graham S. Berninger V. Abbott R. (2012). Are attitudes toward writing and reading separable constructs? a study with primary grade children. *Read. Writ. Q.* 28 51–69. 10.1080/10573569.2012.632732 22736933 PMC3378311

[B25] Guthrie J. T. Wigfield A. Metsala J. L. Cox K. E. (1999). Motivational and cognitive predictors of text comprehension and reading amount. *Sci. Stud. Read.* 3 231–256. 10.1207/s1532799xssr0303_3

[B26] Henk W. A. McKenna M. C. Conradi K. (2011). “Developing affective instrumentation for use in literacy research,” in *Literacy Research Methodologies*, 2nd Edn, eds Duke N. K. Mallette M. H. (New York, NY: Guilford), 242–269.

[B27] Hogsten J. F. Peregoy P. A. (1999). *An Investigation of Reading Attitudes and Self-perceptions of Students Reading on and Below Grade Level.* Available at: https://eric.ed.gov/?q=Hogsten%2c+J.+F.%2c+%26+Peregoy%2c+P.+A.+%281999%29

[B28] Hooper R. (2005). What are teenagers reading? Adolescent fiction reading habits and reading choices. *Literacy* 39 113–120. 10.1111/j.1467-9345.2005.00409.x

[B29] Hume L. E. Lonigan C. J. McQueen J. D. (2015). Children’s literacy interest and its relation to parents’ literacy-promoting practices. *J. Res. Read.* 38 172–193. 10.1111/j.1467-9817.2012.01548.x

[B30] Hutchinson A. Henry L. A. (2010). Internet use and online reading among middle grade students at risk of dropping out of school. *Middle Grades Res. J.* 5 61–75.

[B31] Ivey G. Broaddus K. (2001). Just plain reading: a survey of what makes students want to read in middle school classrooms. *Read. Res. Q.* 36 350–377. 10.1598/RRQ.36.4.2

[B32] Jenkins J. R. Fuchs L. S. van den Broek P. Espin C. Deno L. (2003). Sources of individual differences in reading comprehension and reading fluency. *J. Educ. Psychol.* 95 719–729. 10.1037/0022-0663.95.4.719

[B33] Johnsson-Smaragdi U. Jönsson A. (2006). Book reading in leisure time: long-term changes in young peoples’ book reading habits. *Scand. J. Educ. Res.* 50 519-540. 10.1080/00313830600953600

[B34] Katzir T. Lesaux N. K. Kim Y. S. (2009). The role of reading self-concept and home literacy practices in fourth grade reading comprehension. *Read. Writ.* 22 261–276. 10.1007/s11145-007-9112-8

[B35] Keskin H. K. Bastug M. (2014). A study of the correlations among reading frequency, participation in reading environments and reading attitude. *Int. J. Soc. Sci. Educ.* 4 560–568.

[B36] Kolicì-Vehovec S. Rončevicì Zubkovicì B. Pahljina-Reinicì R. (2014). Development of metacognitive knowledge of reading strategies and attitudes toward reading in early adolescence: the effect on reading comprehension. *Psychol. Top.* 23 77–98.

[B37] Kush J. C. Watkins M. W. (1996). Long-term stability of children’s attitudes toward reading. *J. Educ. Res.* 89 315-319. 10.1080/00220671.1996.9941333

[B38] Lazarus B. D. Callahan T. (2000). Attitudes toward reading expressed by elementary school students diagnosed with learning disabilities. *Read. Psychol.* 21 271–282. 10.1080/027027100750061921

[B39] Ley T. C. Schaer B. B. Dismukes B. W. (1994). Longitudinal study of the reading attitudes and behaviors of middle school students. *Read. Psychol.* 15 11–38. 10.1080/0270271940150102

[B40] Lipps G. (2005). *Faire La Transition: Les Répercussions Du Passage De L’école Primaire À L’école Secondaire Sur Le Rendement Scolaire Et L’adaptation Psychologique Des Adolescents.* Ottawa: Statistique Canada.

[B41] Logan S. Johnston R. (2009). Gender differences in reading ability and attitudes: examining where these differences lie. *J. Res. Read.* 32 199-214. 10.1080/00131911003637006

[B42] Marinak B. A. Gambrell L. B. (2010). Reading motivation: exploring the gender gap. *Lit. Res. Instr.* 49 129–141. 10.1080/19388070902803795

[B43] Martin A. (2008). “Digital literacy and the “digital society,” in *Digital Literacies: Concepts, Policies and Practices*, eds Lankshear C. Knobel M. (New York, NY: Peter Lang), 151–176.

[B44] Martinez R. S. Aricak O. T. Jewell J. (2008). Influence of reading attitude on reading achievement: a test of temporal-interaction model. *Psychol. Schools* 45 1010–1023. 10.1002/pits.20348

[B45] McGeown S. P. Johnston R. S. Walker J. Howatson K. Stockburn A. Dufton P. (2015). The relationship between young children’s enjoyment of learning to read, reading attitudes, confidence and attainment. *Educ. Res.* 57 389–402. 10.1080/00131881.2015.1091234

[B46] McKenna M. C. Conradi K. Lawrence C. Jang B. G. Meyer J. P. (2012). Reading attitudes of middle school students: results of a U.S. Survey. *Read. Res. Q.* 47 283–306. 10.1002/RRQ.021 26181052

[B47] McKenna M. C. Kear D. J. (1990). Measuring attitude toward reading: a new tool for teachers. *Read. Teach.* 43 626–639. 10.1598/RT.43.8.3

[B48] McKenna M. C. Kear D. J. Ellsworth R. A. (1995). Children’s attitudes toward reading: a national survey. *Read. Res. Q.* 30 934–956. 10.2307/748205

[B49] Ministry of Education Recreation and Sports (2011). *Agir Autrement. La Strateìgie d’Intervention Agir Autrement (siaa). Contrer les Eìcarts de Reìussite Entre les Milieux Deìfavoriseìs et Ceux Qui Sont Plus Favoriseìs.* Queìbec, QC: Gouvernement du Queìbec.

[B50] National Center for Education Statistics (2009). *The Nation’s Report Card: Reading 2009: National Assessment of Educational Progress at Grades 4 and 8 (NCES 2010-458).* Washington, DC: Institute of Education Sciences.

[B51] Organisation for Economic Co-operation and Development [OECD] (2015). *Results in focus, Programme for International Student Assessment (PISA).* Available at: http://www.oecd.org/pisa/pisa-2015-results-in-focus.pdf

[B52] Petscher Y. (2010). A meta-analysis of the relationship between student attitudes towards reading and achievement in reading. *J. Res. Read.* 33 335–355. 10.1111/j.1467-9817.2009.01418.x

[B53] Pitcher S. M. Albright L. K. DeLaney C. J. Walker N. T. Seunarinesingh K. Mogge S. (2007). Assessing adolescents’ motivation to read. *J. Adolesc. Adult Lit.* 50 378–396. 10.1598/JAAL.50.5.5

[B54] Sainsbury S. Schagen I. (2004). Attitudes to reading at ages nine and eleven. *J. Res. Read.* 27 373–386. 10.1111/j.1467-9817.2004.00240.x

[B55] Sénéchal M. LeFevre J. (2014). Continuity and change in the home literacy environment as predictors of growth in vocabulary and reading. *Child Dev.* 85 1535–1551. 10.1111/cdev.12222 24467656

[B56] Serbin J. S. Stack D. M. Kingdo D. (2013). Academic success across the transition from primary to secondary schooling among lower-income adolescents: understanding the effects of family resources and gender. *J. Youth Adolesc.* 42 1331–1347. 10.1007/s10964-013-9987-4 23904002

[B57] Swalander L. Taube K. (2007). Influences of family based prerequisites, reading attitude, and self-regulation on reading ability. *Contemp. Educ. Psychol.* 32 206–230. 10.1016/j.cedpsych.2006.01.002

[B58] Unsworth N. McMillan B. D. (2013). Mind wandering and reading comprehension: examining the roles of working memory capacity, interest, motivation, and topic experience. *J. Exp. Psychol.* 39 832–842. 10.1037/a0029669 22905931

[B59] Wolter I. Braun E. Hannover B. (2015). Reading is for girls!? The negative impact of preschool teachers’ traditional gender role attitudes on boys’ reading related motivation and skills. *Front. Psychol.* 6:1267. 10.3389/fpsyg.2015.01267 26379592 PMC4547006

[B60] Worrel F. C. Roth D. A. Gabelko N. H. (2006). Elementary reading attitude survey (ERAS) scores in academically talented students. *Roeper Rev.* 29 119–124. 10.1080/02783190709554395

